# The Homœopathic Eye, Ear, and Throat Journal

**Published:** 1895-03

**Authors:** 


THE HOMŒOPATHIC EYE, EAR, AND THROAT JOURNAL. Editors for eye and ear, A. B. Norton, M. D., and Charles H. Helfrish, M. D.; for nose and throat, John B. Garrison, M. D.; together with fourteen collaborators. Published every month by James A. Robinson, 124 West Eighty-fourth Street, New York. Subscription price, $2.00 per year.

This is a new journal, the first number of which, for January, is just issued. The name sufficiently explains its object. The present number contains accounts of cases treated with the homœopathic remedy. One interesting article is entitled "Eye-strain as a Cause of Epilepsy."

